# Endothelial *Arid1a* deletion disrupts the balance among angiogenesis, neurogenesis and gliogenesis in the developing brain

**DOI:** 10.1111/cpr.13447

**Published:** 2023-03-13

**Authors:** Yuanyuan Wang, Libo Su, Wenwen Wang, Jinyue Zhao, Yanyan Wang, Sihan Li, Yan Liu, Renjie Chai, Xin Li, Zhaoqian Teng, Changmei Liu, Baoyang Hu, Fen Ji, Jianwei Jiao

**Affiliations:** ^1^ State Key Laboratory of Reproductive Biology, Institute of Zoology Chinese Academy of Sciences Beijing China; ^2^ University of Chinese Academy of Sciences Beijing China; ^3^ Innovation Academy for Stem Cell and Regeneration Chinese Academy of Sciences Beijing China; ^4^ School of Life Sciences University of Science and Technology of China Hefei China; ^5^ State Key Laboratory of Reproductive Medicine, School of Pharmacy Nanjing Medical University Nanjing China; ^6^ Institute of Life Sciences, Jiangsu Province High‐Tech Key Laboratory for Bio‐Medical Research Southeast University Nanjing China

## Abstract

The vascular system and the neural system processes occur simultaneously, the interaction among them is fundamental to the normal development of the central nervous system. Arid1a (AT‐rich interaction domain 1A), which encodes an epigenetic subunit of the SWI/SNF chromatin‐remodelling complex, is associated with promoter‐mediated gene regulation and histone modification. However, the molecular mechanism of the interaction between cerebrovascular and neural progenitor cells (NPCs) remains unclear. To generate *Arid1a* ^*cKO‐Tie2*^ mice, *Arid1a* ^*fl/fl*^ mice were hybridized with Tie2‐Cre mice. The Angiogenesis, neurogenesis and gliogenesis were studied by immunofluorescence staining and Western blotting. RNA‐seq, RT‐PCR, Western blotting, CO‐IP and rescue experiments were performed to dissect the molecular mechanisms of Arid1a regulates fate determination of NPCs. We found that the absence of *Arid1a* results in increased the density of blood vessels, delayed neurogenesis and decreased gliogenesis, even after birth. Mechanistically, the deletion of *Arid1a* in endothelial cells causes a significant increase in H3k27ac and the secretion of maternal protein 2 (MATN2). In addition, matn2 alters the AKT/SMAD4 signalling pathway through its interaction with the NPCs receptor EGFR, leading to the decrease of SMAD4. SMAD complex further mediates the expression of downstream targets, thereby promoting neurogenesis and inhibiting gliogenesis. This study suggests that endothelial Arid1a tightly controls fate determination of NPCs by regulating the AKT‐SMAD signalling pathway.

## Graphical abstract

In this study, we found that the absence of chromatin remodelling complex (ARID1A) result in increased the density of blood vessels, delayed neurogenesis and decreased gliogenesis. The loss of endothelial ARID1A leads to a persistent decrease in astrocytes after birth. Mechanistically, the deletion of ARID1A in endothelial cells causes a significant increase in H3k27ac and an increase in the secretion of maternal protein 2 (MATN2). In addition, an increase in endothelial cell‐driven MATN2 alters the Akt/SMAD4 signalling pathway through its interaction with the neural progenitor cells (NPCs) receptor EGFR, leading to the decrease of SMAD4. As a transcriptional activator, SMAD complex mediates the expression of downstream targets, thereby promoting neurogenesis and inhibiting gliogenesis. Taken together, these results suggest that ARID1A tightly controls neurogenesis and gliogenesis by regulating the activity of the AKT‐SMAD signalling pathway.

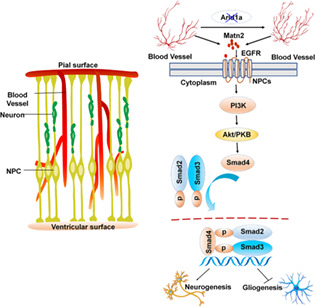

## 1 INTRODUCTION

Mammalian brain development is a complex process that depends on angiogenesis, neurogenesis and gliogenesis.^1^, ^2^, ^3^ Blood vessels support brain function by delivering sufficient oxygen and nutrients.^4^, ^5^ The vascular system and the neural system processes occur simultaneously.^6^, ^7^, ^8^ During embryonic E8.5 to E10.5, meningeal blood vessels gradually penetrate into the developing brain.^9^ The periventricular vessels form a complex network within the telencephalon and gradually spread to the dorsal part of the telencephalon starting from E11.5.^10^ During early embryonic development at E9.5‐E11.5 (mouse), VZ is almost non‐angiogenic. Periventricular vessels regulate progenitor cell behaviour, neurogenesis and gliogenesis. Subsequently, the development of blood vessels is accompanied by changes in gene expression and chromatin epigenetic inheritance.^11^, ^12^ The vascular system coordinates with the nervous system to maintain their homeostasis and participate in the fate of other cells around them. Radial glial progenitors (RGPs) expand the cell pool of the neural set mainly through symmetrical division at E9.5–11.5 (mouse).^13^ Then, the RGP generates multiple types of neurons through asymmetric division, which migrate to the cerebral cortex. At E16.5‐E17.5, RGP enters the gliogenesis stage and produces glial cells. The ordered mitotic behaviour of RGP is the basis of normal brain development. Due to the physical proximity of blood vessels to NPCs, NPCs located in ventricles and sub‐ventricular regions (VZ/SVZ) communicate with blood vessels and receive signals that regulate cell fate determination.^14^, ^15^, ^16^ PI3K/AKT signalling pathway plays a key role in cell proliferation, neuronal differentiation and glial cell differentiation during mammalian cortical development.^17^, ^18^, ^19^ In this process, the vascular system form a homeostasis to guide neural development, for example, by secreting one or more cytokines to change the local microenvironment of NPCs.^20^, ^21^ Therefore, the maintenance of brain homeostasis is mainly achieved through the interaction and coordination between blood vessels and NPCs. In addition, cerebral vessels are also thought to play a role in regulating neuronal activity, glial cell differentiation and apoptotic cell phagocytosis. The development of blood vessels is essential for neurogenesis and gliogenesis. However, the molecular mechanism of cerebrovascular–NPCs interaction remains unclear.

Vascular homeostasis is also associated with chromatin epigenetic inheritance during development. At‐rich interaction domain 1A(Arid1a), a non‐catalytic subunit of the chromosomal remodelling complex, is one of the most commonly mutated genes in tumours and plays a critical role in enhancer mediated gene regulation and chromatin epigenetic inheritance. Arid1a relies on the energy provided by ATP hydrolysis to change the binding position of nucleosome, gene promoter, enhancer and other regions of DNA, remodel chromatin structure, then regulate the expression of genes closely related to multiple cell functions, such as cell replication, differentiation, proliferation and DNA repair. There is evidence showed that Arid1a‐mediated gene regulation plays an important role in anti‐tumour and damage regeneration. For example, inhibition of Arid1a epigenetic modification improves the anti‐tumour activity of CD8T cells,^22^ damages organ regeneration, and leads to organ disorders.^23^, ^24^ In addition, epigenetic factor Arid1a is essential for the establishment of chromatin spatial configuration, and is closely related to cell fate determination, pluripotency maintenance and body development.^25^, ^26^, ^27^ Arid1a regulates the expression of downstream genes by altering the deposition of histone H3K27ac on its promoter/enhancer.^28^, ^29^ Studies have shown that Arid1a, as an epigenetic factor, is involved in vascular remodelling, indicating that Arid1a plays a crucial role in regulating vascular development.^30^ Therefore, it is of great significance to study the function of Arid1a in embryonic cerebrovascular system. However, the underlying molecular mechanism remains unknown.

In this study, we found that the absence of *Arid1a* in blood vessels leads to changes in blood vessel density. Histone modifications alter the chromatin state of homeostasis genes. H3K27ac is a histone marker associated with fate determination. Absence of *Arid1a* leads to increased H3K27ac levels and induces cytokine secretion. Further analysis revealed that maternal protein 2 (Matn2), as a secretory factor, plays a crucial role in signal transduction between blood vessels and NPCs during embryonic brain development. MATN2 secreted by endothelial cells activates the downstream signalling cascade of PI3K by interacting with the neural progenitor receptor EGFR to regulate neurogenesis and gliogenesis during brain development. In summary, our study suggests that Arid1a alters the micro‐environment around NPCs by secreting MATN2 which mediated PI3K/AKT signalling pathway NPCs, further leading to delayed neurogenesis and decreased gliogenesis.

## 2 MATERIALS AND METHODS

### 2.1 Animals

All experimental mice were conducted in accordance with the requirements and specifications of Laboratory Animal Center, Institute of Zoology, Chinese Academy of Sciences. Arid1a floxed (*Arid1a* ^*fl/fl*^)^26^ mouse line was a gift from Hui Lijian Laboratory (Shanghai Institute of Biochemistry and Cell Biology). Tie2‐Cre (TEK‐Cre) mice were purchased from Shanghai model organism. To generate *Arid1a* ^*cKO‐Tie2*^ mice, *Arid1a* ^*fl/fl*^ mice were hybridized with Tie2‐Cre mice. It was confirmed by tail genotype identification and PCR sequencing. All mice were housed at 22–25°C, with a 12‐h light/dark cycle. They were provided adequate food and drinking water.

### 2.2 Isolation and culture of primary mouse endothelial

E15 embryonic brains were rapidly dissected and placed in PBS solution. The meninges are carefully removed from the embryonic brain. The ventral and dorsal sides of the telencephalon were chopped into pieces with a medical blade, transferred into 1.5 mL EP tubes and digested with 1 mL papain at 37°C for 5 min. The sample was shaken regularly every 2 min until digestion is complete. After digestion, the sample was repeatedly blown and ground with a 200 μL pipette gun. The single‐cell suspension was filtered with a sterile 70‐μm nylon mesh (Falcon). The filtered cells were collected and resuspended with 1‐mL erythrocyte lysate at room temperature for 2 min. The lysate was terminated with DMEM (2%FBS) medium. The cells were collected and washed twice with DPBS (Gibco) and labelled by anti‐FITC‐CD31 antibody (Biolegend, 102406). The labelled cells were then sorted by fluorescence activated cell sorting (FACS) AriaIII Fusion (Becton Dickinson). Finally, primary endothelial cells were cultured on 6‐well plates or 24‐well plates (type I collagen Sigma‐Aldrich coated) and cultured on EGM‐2 medium.

### 2.3 Immunofluorescence staining

#### 2.3.1 Antibodies

The following primary antibodies and dilutions were used for Immunostaining and Western blotting anti‐Arid1a (Sigma‐Aldrich, HPA005456); anti‐biotinylated IsolectinB4 (Vector Laboratories, B‐1205); anti‐GFAP (Dako, Z0334); anti‐GFAP (Sigma, G6171); anti‐SOX2 (Cell Signalling Technology, 3728s); anti‐Tbr1 (Abcam; ab31940); anti‐GLAST (Proteintech, 20785‐1‐AP); anti‐S100𝛽 (Abcam, ab52642); anti‐MAP2 (Millipore, MAB3418); anti‐BLBP (Abcam, ab32423); anti‐PDGFR𝛽 (Abcam, ab32570); anti‐CTIP2 (Abcam, ab18465); anti‐SATB2 (Abcam, ab51502); anti‐TUJ1 (Sigma, T2200); anti‐NeuN (Abcam; ab177487); anti‐𝛽‐Actin (Proteintech, 20536‐1‐AP); anti‐𝛽‐Actin (Proteintech; 60008‐1‐Ig); anti‐CD31(BD Biosciences, 553370); anti‐IgG (Bioss; bs‐0295p); anti‐Flag (Sigma, F1804), anti‐HA (Cell Signaling Technology); anti‐Claudin 5 (Invitrogen, 35‐2500). The following florescence secondary antibodies were used: anti‐rabbit Cy3 (Jackson ImmunoResearch), anti‐rat Cy3 (Jackson ImmunoResearch), anti‐mouse Cy3 (Jackson ImmunoResearch), anti‐rat Alexa Fluor 488 (Jackson ImmunoResearch), anti‐rabbit Alexa Fluor 488 (Jackson ImmunoResearch), anti‐goat Alexa Fluor 488 (Jackson ImmunoResearch).

### 2.4 Isolation and culture of primary mouse NPC

Minor modifications were made to the isolation and culture of primary mouse NPCs. E14 or E15 embryonic brains were rapidly dissected and placed in PBS solution. The meninges were carefully removed with tweezers and transferred into 1.5‐mL tubes. Digest with 1‐mL Papain at 37°C for 5 min. Shake every 2 min until digestion is complete. Then, the sample was repeatedly blown with a 200‐μL pipette gun and the single‐cell suspension was filtered with a sterile 70‐μm nylon mesh (Falcon). Finally, primary neural precursor cells were cultured on 6‐well or 24‐well plates coated with laminin and poly (d‐lysine).

### 2.5 Three‐dimensional reconstruction

Confocal imaging of 40 μm brain sections was performed on a Zeiss LSM880 microscope. The vessels were scanned from top to bottom at 0.83 μm intervals in the Z direction. Images were analysed using Imaris 9.0 software. Subsequently, Surface module in Imaris 9.0 software was used to measure the surface area of blood vessels, and Filament module was used to measure the volume of blood vessels. The results were used for vascular density analysis and three‐dimensional (3D) reconstruction.

### 2.6 RNA‐sequencing and data analysis

Total RNA of E15 cortex was extracted from primary endothelial cells isolated by FACS using the RNAeasy Mini kit (QIAGEN). RNA quality was analysed by Agilent 2100 Bioanalyzer. The sequencing was performed by Anoroad using Illumina HiSeq 2500 platform. All sequencing data reported in this paper were submitted to NCBI's GEO with accession number GSE221176.

### 2.7 Plasmid preparation

Mouse *Matn2* cDNA was constructed into pCDH‐3Flag vector and *Egfr* cDNA was subcloned into the pCDH‐3HA vector. *Matn2 shRNA* were cloned into the pSicoR‐GFP vector. *Matn2‐shRNA1*: CATGCTCAAAGATTGACTATT; *Matn2‐shRNA2*: GCAAAGGTCAAGGAGTTCATT.

### 2.8 Co‐immunoprecipitation

The sample was cleaved in RIPA (Solarbio) with a 1% protease inhibitor cocktail and 1%PMSF. The supernatant was collected by centrifugation at 4°C and incubated overnight with 25‐μL anti‐Flag or anti‐HA magnetic beads (MBL) at 4°C. After washing for three times and boiling for 10 min, the protein was analysed by western blotting.

### 2.9 Co‐culture

NPCs were laid on the coverslips coated with poly‐d‐Lysine and Laminin were placed in the bottom chamber of a transwell plate, endothelial cells were then seeded in the top chamber. The virus was added by equal volume after 24 h. Cells were cultured for 3 days and processed for immunocytochemistry as described earlier.

### 2.10 Statistical analysis

All immuno‐stained sections were imaged using Zeiss LSM880. During 3D vascular reconstruction, 40‐μm brain sections were used and Imaris 9.0 software was used to generate images for statistical purposes. The Filament module in Imaris 9.0 software analyses the density of blood vessels. All statistical analyses were performed using GraphPad Prism software 6.0. Unpaired two‐tailed Student's *t*‐test was used between the two groups, and one‐way ANOVAs were used for multiple comparisons. The results are expressed as mean ± SEM. *p < 0.05, **p < 0.01, ***p < 0.001, n.s., not significant.

## 3 RESULTS

### 3.1 The developing blood vessels and neural progenitor cells are well‐positioned to interact in vivo

**FIGURE 1:**
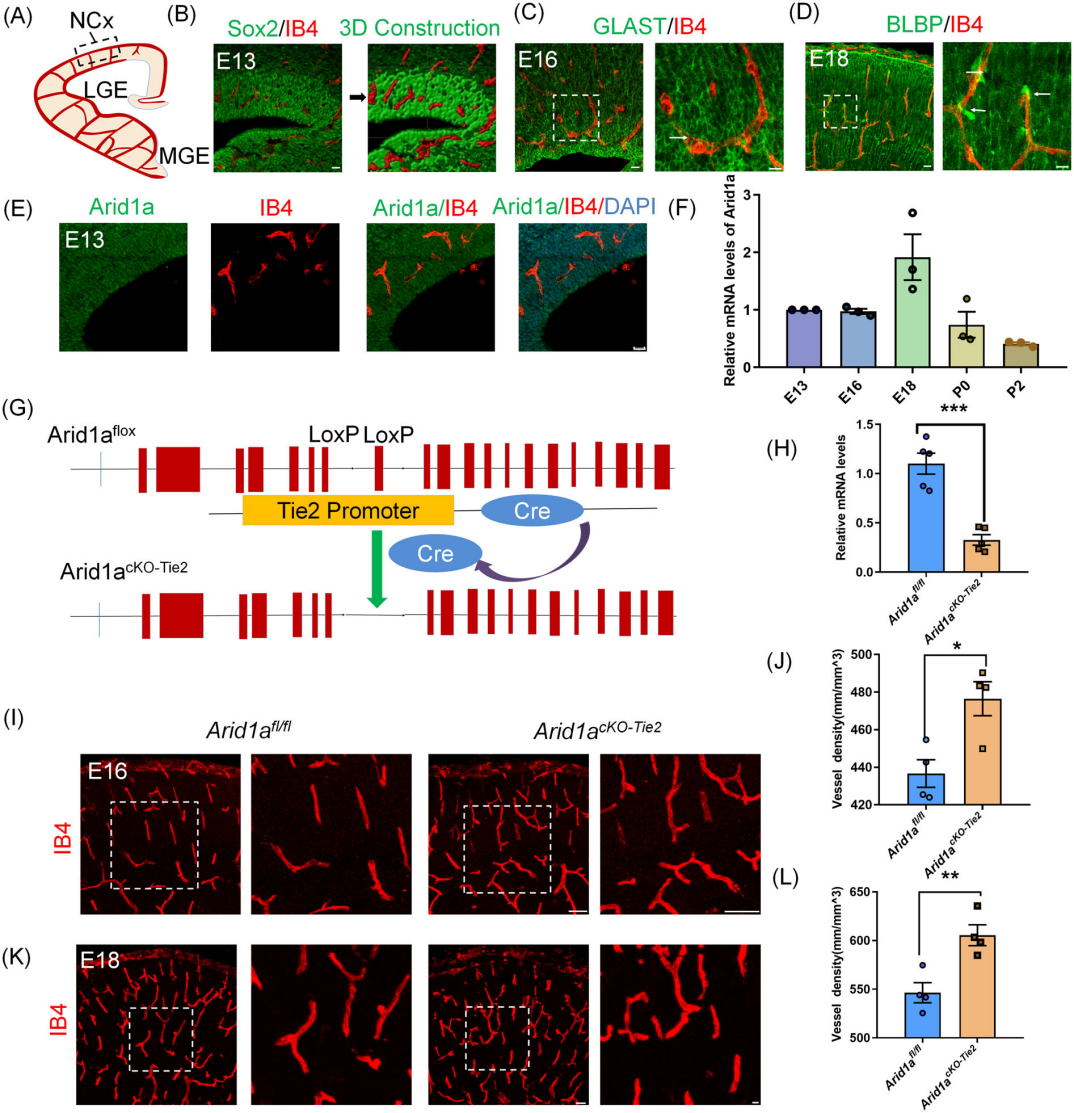
Endothelial contact specialized areas of neural progenitor cells (NPCs) and *Arid1a* deletion disrupts the vascular homeostasis. (A) Diagram of the vascularization pattern in the mouse cerebral cortex. NCx neocortex, LGE lateral ganglionic eminence, MGE medial ganglionic eminence. (B) Immunofluorescence staining of Sox2 and IB4 at E13 in the embryonic neocortex. SOX2, the neural progenitor marker; IB4, the blood vessels marker. The right is three‐dimensional reconstructions of z‐stacks in brain vessels and NPCs. Scale bars, 20 μm. (C) The blood vessels are located near astrocyte precursor cells (APCs). Immunofluorescence staining of GLAST and IB4 at E16 in the embryonic neocortex. GLAST, the astrocyte precursor cells. The right shows an enlarged image of the delineated area. Scale bars, 20 μm (left), 10 μm (right). (D) The blood vessels are located near intermediate astrocyte precursor cells (APCs). Immunofluorescence staining of BLBP and IB4 at E18 in the embryonic neocortex. BLBP, the astrocyte precursor cells. The right is shown an enlarged image of the delineated area. Scale bars, 20 μm (left), 10 μm (right). (E) Arid1a is highly expressed in blood vessels. Confocal images of IB4 and Arid1a at E13 immunostaining in mouse brain section. Scale bars, 20 μm. (F) Relative mRNA levels of *Arid1a* in brain endothelial at E13, E16, E18, P0 and P2, *n* = 3 each group. (G) Schematic construction of endothelial cell *Arid1a* conditional knockout mice. LoxP sites were inserted into both ends of exon 8 in these mice. Cre is an endothelium‐specific recombinant enzyme. (H) Quantitative RT‐PCR analysis of relative *Arid1a* mRNA abundance in the *Arid1a* ^*fl/fl*^ and *Arid1a* ^*cKO‐Tie2*^ brains purified endothelial, *n* = 5 each group. (I) Confocal images of IB4 staining showed the increased vascular density in the mutant's cortex at E16. The right shows an enlarged image of the delineated area. Scale bars, 20 μm (left), 20 μm (right). (J) Quantification of the blood vessel density showing a disruption in *Arid1a* ^*cKO‐Tie2*^ brain cortex at E16, *n* = 4 each group. (K) IB4 immunostaining revealed a persistent disruption of endothelial at E18. The right shows an enlarged image of the delineated area. Scale bars, 100 μm (left), 50 μm (right). (L) Quantification of the density of blood vessel showing a persistent disruption in *Arid1a* ^*cKO‐Tie2*^ brain cortex at E18, *n* = 4 each group. The data are represented as the mean ± SEM. Unpaired two‐tailed Student's *t*‐test, one‐way ANOVA; *p < 0.05, **p < 0.01, ***p < 0.001.

In the early stage of brain development, neural development and vascular development occur simultaneously.^6^, ^7^, ^31^ Blood vessels act as niches and scaffolds for the migration of neurons during development. The establishment of vascularization in the central nervous system (CNS) is essential to maintain the homeostasis of neural networks.^32^ We first examined the developmental patterns of blood vessel and neural progenitor cells (NPCs) during the embryonic period (E13‐P0), which is a critical period for the fate determination of NPCs. We first examined the position relationships of blood vessels and neural precursor cells at E13 embryo. The results shows that the vascular colonization was in the VZ/SVZ region and was very close to the physical location of SOX2^+^ neural stem cells (Figure 1A,B). We also found that the physical positions of blood vessels and glial progenitor cells (GLAST and BLBP) at E16 and E18 were very close (Figure 1C,D; Figure S1A). Similar phenomena appeared at P0 (Figure S1B). These data suggest that developing blood vessels are closely related to neural precursor cells and may play a crucial role in regulating the fate determination of cortical NPCs. In addition, we assessed the expression of Arid1a in endothelial cells and found that Arid1a was highly expressed in endothelial cell (Figure S1C). In order to further explore the expression of Arid1a during cerebrovascular development, we also performed immunostaining on E13.5 blood vessels. Results showed that Arid1a was highly expressed at E13.5, suggesting that Arid1a may play an important role in early brain development (Figure 1E). In addition, epigenetic inheritance is related to vascular development. Therefore, it will be important to determine whether Arid1a also plays a role in vascular development. To answer this question, we purified ECs by FACS and performed RT‐PCR using Arid1a‐specific primers. The results showed that the expression of Arid1a gradually changed from day E13 to P2 (Figure 1F), indicating that the expression of Arid1a in ECs are correlated with the fate determination of neural precursor cells during embryonic development.

### 3.2 Loss of epigenetic *Arid1a* increases the density of blood vessels

To investigate whether the loss of *Arid1a* in endothelial alters vascular remodelling, we crossed the Tie2‐Cre mouse lines with mice carrying the *Arid1a* conditional allele (*Arid1a* ^*fl/fl*^) to generate endothelial conditional knockout mice (*Arid1a* ^*cKO‐Tie2*^) (Figure 1G). *Arid1a* ^*cKO‐Tie2*^ mice exhibit a specific deletion of endothelial cells *Arid1a* during cortical development. First, endothelial cells purified by FACS were cultured to identify the absence of *Arid1a* expression in the vessels of the mutated cerebral cortex (Figure S1D). Western blotting results showed that *Arid1a* was specifically knocked out in blood vessels (Figure S1E). In addition, RT‐PCR analysis of endothelial cells confirmed a decrease in the abundance of *Arid1a* mRNA in the cerebral cortex of *Arid1a* ^*cKO‐Tie2*^ (Figure 1H).

Next, we detected changes in vascular homeostasis at different stages of cortical neurogenesis and gliogenesis in *Arid1a* ^*fl/fl*^ and *Arid1a* ^*cKO‐Tie2*^ by immunostaining IB4. These results suggest that epigenetic *Arid1a* loss impairs vascular homeostasis at E16 and E18, at which point neurogenesis and gliogenesis have begun. The *Arid1a^cKO‐Tie2^* had significantly higher vascular density than *Arid1a* ^*fl/fl*^ at E16 (Figure 1I,J). In addition, the immunostaining results of E18 were similar (Figure 1K,L). Taken together, these results suggest that epigenetic Arid1a loss increase cerebral vascular density and disrupt vascular homeostasis in developing cortex. In addition, immunofluorescence staining showed that endothelial *Arid1a* depletion did not affect perivascular recruitment and tight connections (Figure S2A–D). We used the fluorescent tracer Alexa Fluor 555 cadaver amine (Cad‐A555) to detect blood–brain integrity and found no significant blood–brain barrier leakage in the cortex of *Arid1a* ^*cKO‐Tie2*^ mice (Figure S2E). These results suggest that the loss of endothelial *Arid1a* has no effect on the blood–brain barrier.

### 3.3 Loss of endothelial cells *Arid1a* disrupts neurogenesis and gliogenesis during brain development

**FIGURE 2:**
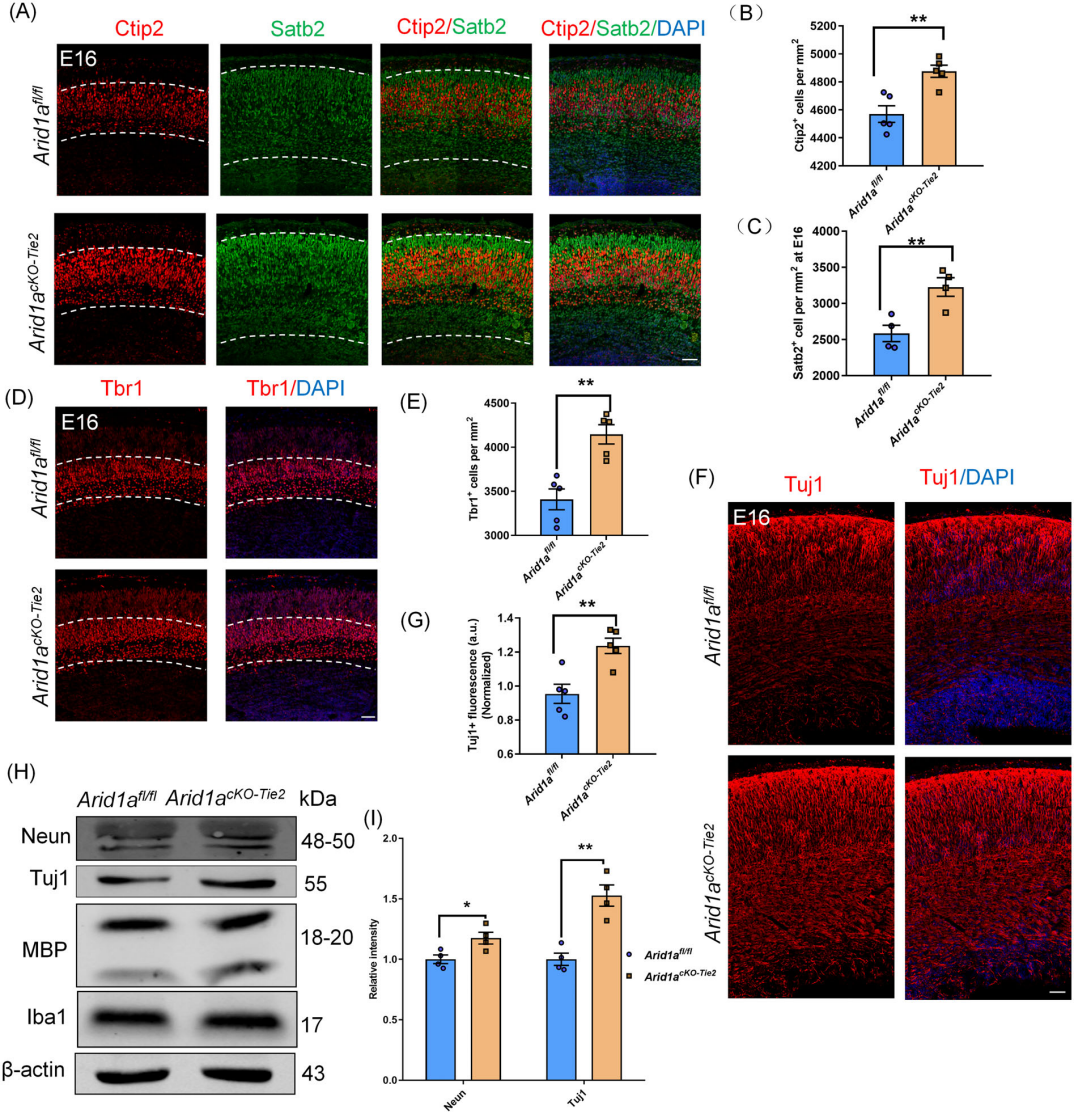
Neurons are increased in mutant cortex. (A) Confocal immunofluorescence image of Ctip2 and Satb2 in *Arid1a* ^*cKO‐Tie2*^ at E16. Scale bars, 50 μm. (B) Quantification of increased number of Ctip2^+^ neurons in *Arid1a* ^*cKO‐Tie2*^ cortex, *n* = 5 each group. (C) Quantification of increased number of Satb2^+^ neurons in *Arid1a* ^*cKO‐Tie2*^ cortex, *n* = 4 each group. (D) Confocal immunofluorescence image of Tbr1 in *Arid1a* ^*cKO‐Tie2*^ cortex at E16. Scale bars, 50 μm. (E) Quantification of increased number of Tbr1^+^ neurons in *Arid1a* ^*cKO‐Tie2*^ cortex, *n* = 5 each group. (F) Confocal immunofluorescence image of Tuj1 revealed an increase of total neurons in *Arid1a* ^*cKO‐Tie2*^ cortex at E16. Scale bars, 50 μm. (G) Quantification of increased number of Tuj1^+^ neurons in *Arid1a* ^*cKO‐Tie2*^ cortex, *n* = 5 each group. (H) Western blot analysis of the expression levels of neuron markers Tuj1 and Neun in *Arid1a* ^*cKO‐Tie2*^ mice cortex. (I) Quantification of Tuj1 and Neun expression levels in *Arid1a* ^*cKO‐Tie2*^ mice cortex, *n* = 4 each group. The data are represented as the mean ± SEM. Unpaired two‐tailed Student's *t*‐test, one‐way ANOVA; *p < 0.05, **p < 0.01.

**FIGURE 3:**
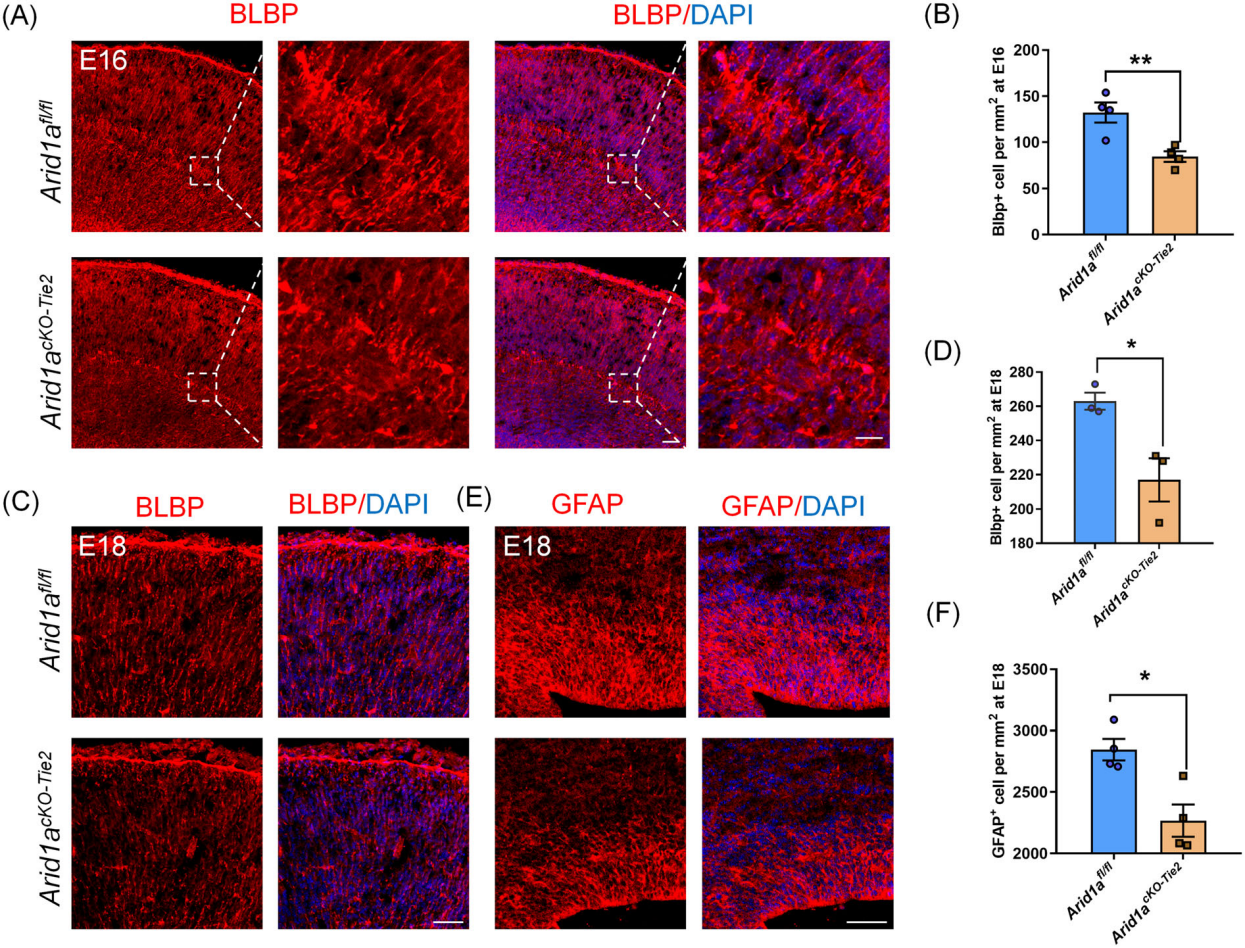
Glial precursors are decreased in mutant cortex. (A) Confocal immunofluorescence image of BLBP revealed a decrease in *Arid1a* ^*cKO‐Tie2*^ cortex at E16. Scale bars, 50 um (left) 20 μm (right). (B) Quantification of decreased number of BLBP^+^ cell in *Arid1a* ^*cKO‐Tie2*^ cortex, *n* = 4 each group. (C) Confocal immunofluorescence image of BLBP revealed a decrease in *Arid1a* ^*cKO‐Tie2*^ cortex at E18. Scale bars, 50 μm. (D) Quantification of decreased number of BLBP+ cell in *Arid1a* ^*cKO‐Tie2*^ cortex, *n* = 3 each group. (E) Confocal immunofluorescence image of GFAP revealed a decrease in *Arid1a* ^*cKO‐Tie2*^ cortex at E18. Scale bars, 50 μm. (F) Quantification of decreased number of GFAP^+^ cell in *Arid1a* ^*cKO‐Tie2*^ cortex, *n* = 4 each group. Data are represented as means ± SEM. unpaired two‐tailed Student's *t*‐test, one‐way ANOVA; *p < 0.05, **p < 0.01.

During brain development, the orderly generation of neurons and glial cells is essential for the structure and function of the cerebral cortex. This process requires the micro‐environment to precisely coordinate the fate of the NPCs.^33^ Given the increased vascular density in the mutated cortex, we evaluated whether neuronal generation in the cerebral cortex was impaired when vascular homeostasis was disrupted. First, we detected the changes of neurons in each layer by immunostaining Ctip2 and Satb2. The results showed that the number of Ctip2^+^ and Satb2^+^ neurons are increased in the mutant cortex (Figure 2A–C). Similar results were found in deep Tbr1^+^ neurons in the mutated cortex (Figure 2D,E). Then, we proceeded to use the immunostaining Tuj1 to detect changes in neurons that are generally marked in the cortex. The results showed that there is an increase of Tuj1^+^ neurons in the mutant cortex (Figure 2F,G). Western blotting also confirmed that the expression of Neun and Tuj1 increased when *Arid1a* were deprived in endothelial cells (Figure 2H,I). On the other hand, we performed a series of immunostaining assays to detect changes in gliogenesis. we observed a decrease in the number of brain lipid‐binding protein (BLBP^+^) glial progenitor cells in *Arid1a* ^*cKO‐Tie2*^ mice at E16 (Figure 3A,B). Similar results were found for the number of BLBP^+^ glial progenitor cells, GFAP^+^ glial progenitor cells and GLAST^+^ astrocytes in the mutant cortex (Figure 3C–F; Figure S3A,B) at E18. A decrease in the number of GFAP^+^ astrocyte and Glutamine synthetase (GS^+^) glial cells were also observed at p0 (Figure S3C–F). We hypothesized that endothelial *Arid1a* depletion may be an obstacle to gliogenesis. To explore the potential effect of apoptosis on cell fate determination in NPCs, we performed a dUTP nickel end labelling assay (TUNEL) mediated by terminal deoxynucleotide transferase to detect apoptosis. The results showed that there is no significant difference between *Arid1a* ^*fl/fl*^ and *Arid1a* ^*cKO‐Tie2*^ cortical cell apoptosis (Figure S3G,H), confirming that the increase in the number of neurons and the decrease in the number of glial progenitor cells are not caused by apoptosis. Besides, we detected the effect of *Arid1a* deletion in endothelial cells on the proliferation of NPCs, the results showed no difference between *Arid1a* ^*fl/fl*^ and *Arid1a* ^*cKO‐Tie2*^ cortical proliferation of NPCs (Figure S3I,J). These results suggested that Arid1a in endothelial cells plays an important role in regulating fate determination of NPCs and that *Arid1a* knockout can promote neurogenesis and inhibit gliogenesis.

### 3.4 Loss of epigenetic *Arid1a* leads to persistent decrease of astrocyte production and increase of neuron production during the developing cortex

**FIGURE 4:**
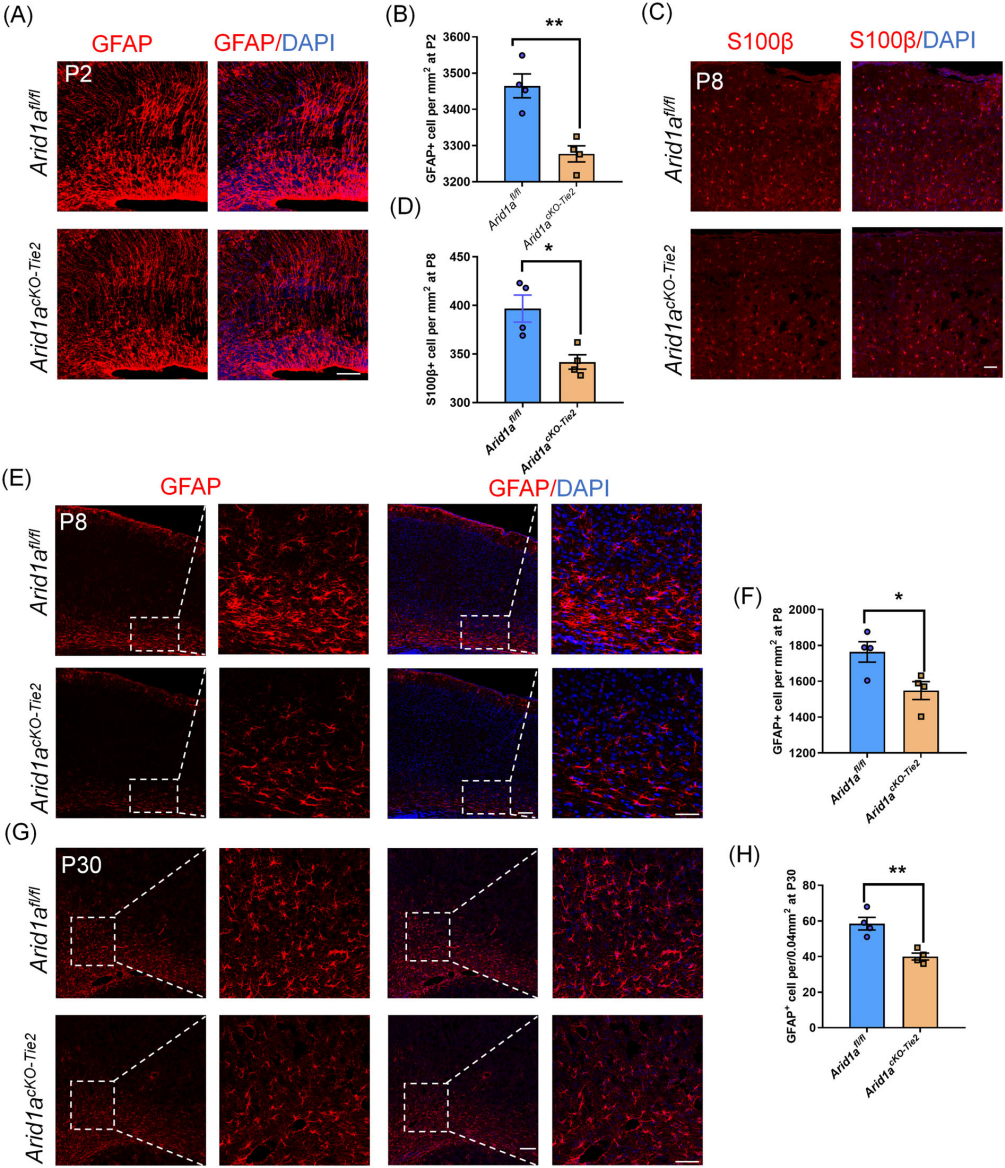
Astrocyte decreased in mutant cortex. (A) Confocal immunofluorescence image of GFAP revealed a decrease in *Arid1a* ^*cKO‐Tie2*^ cortex at P2. Scale bars, 50 μm. (B) Quantification of decreased number of GFAP^+^ cell in *Arid1a* ^*cKO‐Tie2*^ cortex, *n* = 4 each group. (C) Confocal immunofluorescence image of S100β^+^ revealed a decrease in *Arid1a* ^*cKO‐Tie2*^ cortex at P8. Scale bars, 50 μm. (D) Quantification of decreased number of S100β^+^ cell in *Arid1a* ^*cKO‐Tie2*^ cortex, *n* = 4 each group. (E) Confocal immunofluorescence image of GFAP revealed a decrease in *Arid1a* ^*cKO‐Tie2*^ cortex at P8 stage. Scale bars, left (100 μm), right (50 μm). (F) Quantification of decreased number of GFAP^+^ cell in *Arid1a* ^*cKO‐Tie2*^ cortex at P8, *n* = 4 each group. (G) Confocal immunofluorescence image of GFAP revealed a decrease in *Arid1a* ^*cKO‐Tie2*^ cortex at P30 stage. Scale bars, left (100 μm), right (50 μm). (H) Quantification of decreased number of GFAP^+^ cell in *Arid1a* ^*cKO‐Tie2*^ cortex at P30, *n* = 4 each group. Data are represented as means ± SEM. Unpaired two‐tailed Student's *t*‐test, one‐way ANOVA; *p < 0.05, **p < 0.01.

We examined long‐term astrocyte production after birth. At P2, the number of GFAP^+^ astrocytes in *Arid1a* ^*cKO‐Tie2*^ mice were significantly lower than in *Arid1a* ^*fl/fl*^ mice (Figure 4A,B). In addition, we observed that the number of GS^+^ and S100β^+^ astrocytes in *Arid1a* ^*cKO‐Tie2*^ mice were also obviously lower than in *Arid1a* ^*fl/fl*^ mice (Figure S4A–D).

At P8, we observed a decrease in the number of S100β^+^ and GFAP^+^ positive astrocytes (Figure 4C–F). At P30, we detected a sustained decrease in the number of GFAP^+^ positive astrocytes (Figure 4G,H). These results showed a sustained decrease in astrocyte production in the cortex of endothelial *Arid1a* knockout mice.

On the other hand, we examined postnatal neurogenesis after birth. At P8, the number of NEUN^+^ neurons in *Arid1a* ^*cKO‐Tie2*^ mice were significantly higher than in *Arid1a* ^*fl/fl*^ mice (Figure S4E,F). These results indicate a sustained increase in neurogenesis in the cortex of endothelial *Arid1a*‐deficient mice.

### 3.5 The Absence of *Arid1a* disrupts histone modification in embryonic endothelial cells, then which regulates the fate determination of NPCs by secreting MATN2

**FIGURE 5:**
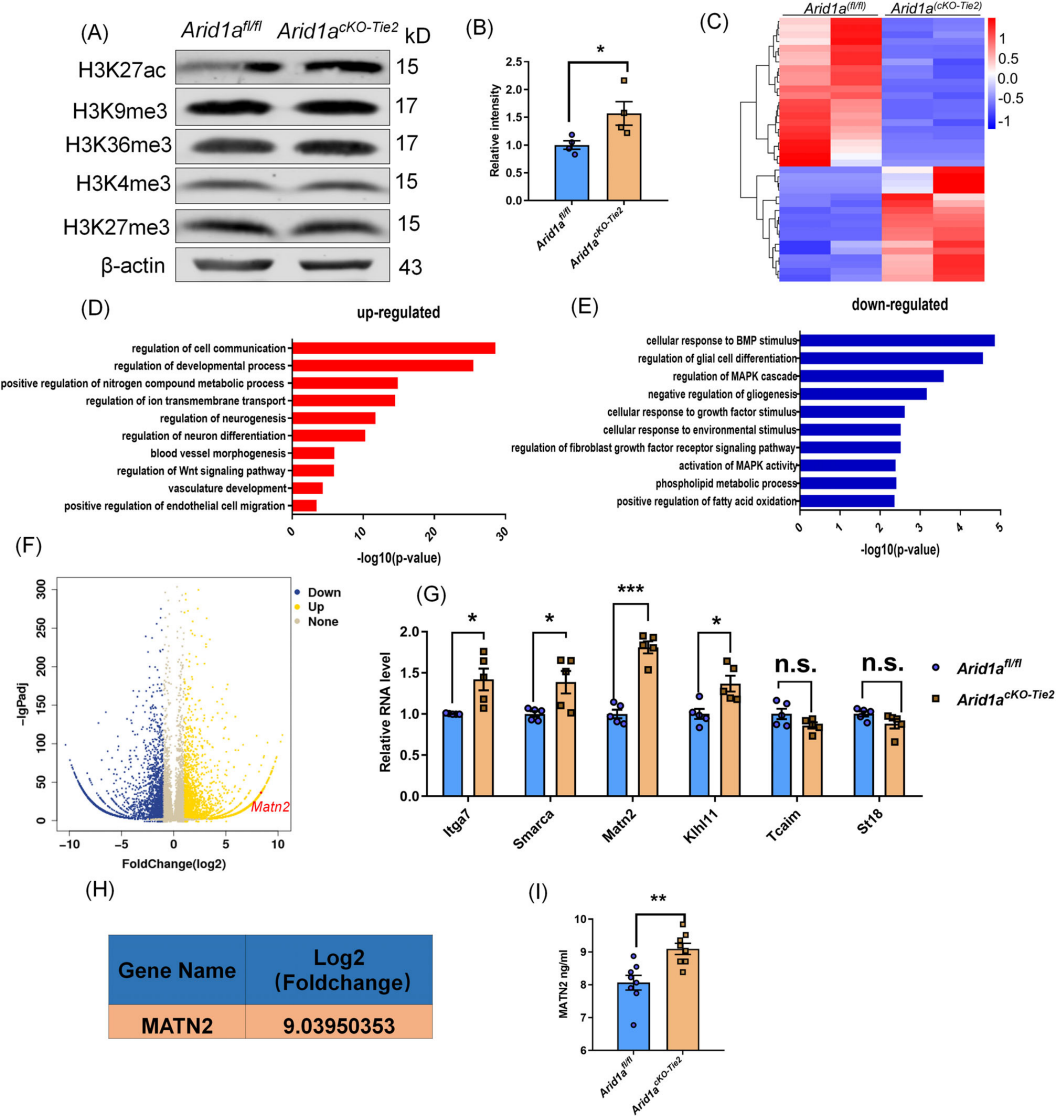
The changing in histone modification of endothelial cells regulate the fate determination of neural progenitor cells by targeting secretory Matn2. (A) Western blotting results showed that the expression level of H3K27ac in endothelial cells increased with *Arid1a* deletion. (B) Quantification of H3K27ac expression levels in endothelial, n = 4 each group. (C) Heat maps showed many differentially expressed genes in purified endothelial cell RNA‐seq data. Gene Ontology (GO) analysis of cell communication related to the up‐regulated (D) and down‐regulated (E) genes in the *Arid1a*‐depleted endothelial by RNA‐seq. (F) Volcano plots showing differentially expressed genes between *Arid1a* ^*fl/fl*^ and *Arid1a* ^*cKO‐Tie2*^ endothelial in RNA‐seq data. (G) Six different genes in mouse cortical ECs were analysed by qPCR, among which Matn2 had the most significant difference, *n* = 5 each group. (H) The Matn2 gene was one of the significantly differentially expressed secretory genes in Endothelial. (I) The expression of Matn2 in cultured endothelial cells was detected by ELISA, *n* = 8 each group. The data are represented as the mean ± SEM. Unpaired two‐tailed Student's *t*‐test, one‐way ANOVA; *p < 0.05, **p < 0.01, ***p < 0.001.

Histone modification of endothelial cells can reveal information about the regulatory mechanisms of changes in gene expression. Arid1a has been associated with nucleosome composition, histone modification and fate determination.^27^, ^34^, ^35^ We examined a range of histone modifications, including H3K4me3, H3K9me3, H3K27me3, H3K27ac, and H3k36me3, where H3K27ac was significantly increased in *Arid1a*‐deficient endothelial cells (Figure 5A,B). These results not only reveal the effect of *Arid1a* deletion in endothelial cells on histone modification, but also indicate that Arid1a plays an important role in vascular homeostasis. To explore the regulation of H3K27ac on specific gene expression, we performed high‐throughput RNA‐seq sequencing on endothelial cells. First, heat maps of transcripts were generated (Figure 5C). By comparing gene expression in *Arid1a* ^*fl/fl*^ and *Arid1a* ^*cKO‐Tie2*^ endothelial cells, gene ontology analysis results showed that up‐regulated gene enrichment was involved in cell communication, intracellular signal transduction, vascular development and neuronal differentiation (Figure 5D). Down‐regulated genes are involved in gliogenesis, phospholipid metabolism and fatty acid oxidation (Figure 5E). Next, we measured differences in gene expression in endothelial cells. The maternal protein Matn2 gene was significantly increased, which was consistent with the volcanic map and RT‐PCR results (Figure 5F–H). The increase of Matn2 gene was significantly related to the fate determination of NPC. In addition, MATN2 concentrations in the supernatant of *Arid1a* ^*fl/fl*^ and *Arid1a* ^*cKO‐Tie2*^ cortical purified endothelial cells were measured. The results showed that the concentration of Matn2 in *Arid1a*‐deficient endothelial cells was significantly higher than that in normal endothelial cells (Figure 5I). At the same time, we examined the expression of Matn2 in endothelial by immunostaining. The result shows that the expression of *Matn2* in *Arid1a* ^*cKO‐Tie2*^ mice are significantly higher than that in *Arid1a* ^*fl/fl*^ mice (Figure S5B). In conclusion, the RNA‐seq results strongly suggested that *Arid1a* deletion in endothelial cells leads to abnormal genome‐wide deposition of H3K27ac, thereby enhancing transcriptional activity and regulating *Matn2* gene expression. During embryonic brain development, endothelial cells secrete MATN2 as a direct downstream target to participate in NPC response.

### 3.6 Endothelial cell‐driven MATN2 is involved in the fate of NPCs through AKT‐SMAD signal transduction

It is well known that endothelial cells and NPCs have the same spatial and temporal interval, vascular development occurs simultaneously with neural development, which can promote neurogenesis and support the brain development and physiological functions.^21^, ^36^, ^37^ Endothelial cells not only play a role in brain development, but also are crucial in the fate determination of NPCs. To explore the molecular signalling mechanisms by which endothelial cells regulate the fate determination of NPCs, we performed KEGG pathway analysis on the RNA‐seq dataset and identified potential responses involved in signalling pathways which regulate stem cell pluripotency in brain development (Figure S5A).

**FIGURE 6:**
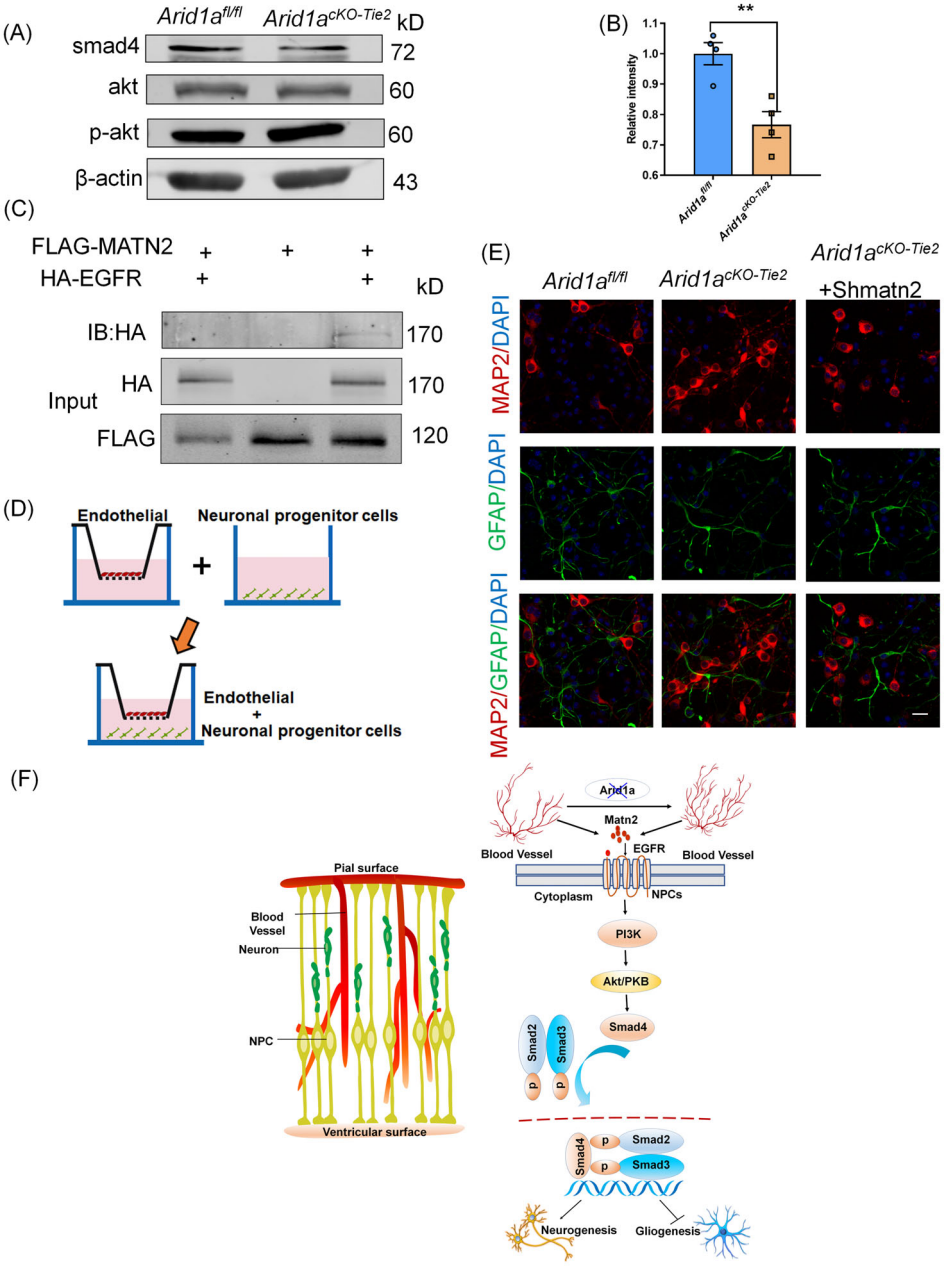
Vascular homeostasis regulated the fate determination of neural progenitor cells (NPCs) through the activation of PI3K/AKT signalling. (A) Western blotting analysis the expression levels of Smad4 and P‐akt in *Arid1a* ^*fl/fl*^ and *Arid1a* ^*cKO‐Tie2*^ embryonic cortex. (B) Quantification of Smad4 and P‐akt expression levels in *Arid1a* ^*cKO‐Tie2*^ mice cortex, *n* = 4 each group. (C) Co‐IP experiments were performed to test the interaction between MATN2 and EGFR in 293FT cells. (D) Schematic diagram of co‐culture of neural precursor cells (NPCS) and endothelial cells. (E) Confocal immunofluorescence images showed that endothelial cell *Matn2* knockdown could rescue developmental abnormalities caused by endothelial cell *Arid1a* deletion. Scale bars, 20 μm. (F) A model showing how endothelial cells regulate the fate of neural progenitor cells in the developing brain. The data are represented as the mean ± SEM. Unpaired two‐tailed Student's *t*‐test, one‐way ANOVA; **p < 0.01.

In addition, key pathways involved in PI3K/AKT signalling induced changes in gene expression also play a number of key roles in the fate determination of NPCs. Therefore, NPCs of VZ/SVZ regions were isolated from the *Arid1a* ^*fl/fl*^ and *Arid1a* ^*cKO‐Tie2*^ cerebral cortex to detect whether AKT signalling was affected in the NPCs. Levels of proteins associated with signalling pathways were detected and showed increased phosphorylation of AKT and decreased binding partner SMAD4(Figure 6A,B). This SMAD complex transfers to the nucleus and regulates gene expression. The results showed that the disruption of vascular homeostasis disturbs the transportation of the SMAD complex into the nucleus, thus regulating the fate determination of NPCs, which was also consistent with previous reports.^38^

How does endothelial cell‐derived Matn2 trigger the AKT‐SMAD signalling pathway to regulate NPCs fate determination? MATN2 carries several EGF‐like domains.^39^ It is speculated that MATN2 as a decoy molecule may interact with EGFR. In order to verify whether MATN2 interacts with EGFR to activate the downstream cascade signalling of the SMAD pathway, co‐immunoprecipitation tests were conducted. The result showed that flag‐labelled MATN2 pulled down HA‐labelled EGFR, indicating a direct interaction between MATN2 and EGFR (Figure 6C). In conclusion, endothelial cell‐driven MATN2 activates the PI3K/ AKT/SMAD signalling pathway by interacting with the EGFR receptor during brain development, thereby regulating NPCs fate determination. To further investigate whether changes in NPCs fate determination caused by *Arid1a* deletion in endothelial cell can be rescued by inhibiting MATN2, we first constructed a mouse *Matn2* short hairpin RNA (shRNA) plasmid and verified the knockdown efficiency of *Matn2‐shRNA* at mRNA level in endothelial (Figure S5C). In addition, we introduced *Matn2‐shRNA* into an in vitro co‐culture system of NPCs and endothelial cells. Primary endothelial cells were co‐cultured with NPCs in vitro and treated with exogenous *Matn2‐shRNA* virus for 3 days. Immunostaining showed that the proportion of MAP2^+^ neurons in the *Arid1a* ^*cKO‐Tie2*^ group decreased after *Matn2‐shRNA* stimulation (Figure 6D,E), which is similar to that in the *Arid1a* ^*fl/fl*^ group, suggesting that endothelial *Matn2* knockdown could rescue the developmental imbalance caused by endothelial *Arid1a* deletion. Taken together, our study sheds light on the interaction between endothelial cells and NPC responses. Loss of *Arid1a* disrupts vascular homeostasis by altering histone modification, leading to an increase in MATN2, which communicates signals to NPCs by interacting with EGFR receptors, thereby regulating fate determination of NPCs (Figures 6F and S5D,E).

## 4 DISCUSSION

The complexity of embryonic brain development not only involves the ability of NPC to generate neurons and glial cells, but also involves the interaction between multiple systems to ensure the precise regulation of tissue homeostasis function. The vascular system and the nervous system develop almost simultaneously, providing an ideal model for studying the interaction of the vascular and microenvironment during brain development. Our study shows that epigenetic Arid1a mediates H3K27ac levels in endothelial cells, regulates gene expression and vascular homeostasis. Loss of *Arid1a* in endothelial cells increases vascular density, delays neurogenesis and reduces gliogenesis. Disruption of vascular homeostasis alters environmental signals and the fate determination of NPCs. In this study, we describe a novel biological mechanism whereby endothelial cells play an important role in regulating NPCs fate determination through the release of cytokines during embryonic brain development.

Arid1a is one of the subunits of chromatin remodelling complex SWI/SNF with DNA binding sites. Deletion of Arid1a will destroy the gliding activity of nucleosomes on chromosomes, change histone modification in promoters, enhancers and regulate the expression of downstream genes.^28^, ^40^, ^41^ Consistent with previous findings, Arid1a regulates gene expression by remodelling histone modifications in endothelial cells. Our western blotting results showed that *Arid1a* knockout significantly increased H3K27ac histone modification in endothelial cells and led to changes in cytokine related neurogenic pathways. The expression of the directly secreted cytokine MATN2 was determined by using the high‐throughput RNA‐seq data set of endothelial cells and information of up‐regulated genes.

Fate determination of NPCs in embryonic cortex requires the regulation of transcription factors and cytokines secreted by surrounding cells. Cytokines contain a variety of proteins that coordinate signal exchange between different tissues, organs and cells, further regulate the fate determination of NPCs.^42^, ^43^ Arid1a‐mediated histone modification is associated with gene expression levels, impairs vascular homeostasis and regulates cytokine release in the CNS. Endothelial cell‐driven MATN2 is capable of interacting with multiple receptor families and plays an important role in the differentiation and regeneration of myogenic and nerve tissues. Previous studies have shown that various signalling pathways, such as Wnt/β‐catenin, Notch cascade and bone morphogenetic protein (BMP), play a crucial role in regulating the fate determination of NPCs during the development of embryonic cortex.^44^, ^45^, ^46^ It is worth noting that BMP pathway is crucial for embryonic brain development and microenvironment homeostasis, including maintenance of neural differentiation and signal transduction in the micro‐environment.^47^, ^48^ Epidermal growth factor receptor (EGFR) is a co‐receptor initiating downstream activation, especially involved in the signal transduction of typical BMP.^49^, ^50^ EGFR signalling pathway plays an important role in the physiological processes of cell growth, proliferation and differentiation. We found that during embryonic brain development, MATN2 were secreted by endothelial cells interact with EGFR receptors on the surface of NPCs to further regulate the fate determination of NPCs. Consistent with previous studies, EGFR is largely expressed in NPCs and is involved in signal transduction.^51^, ^52^ The interaction between MATN2 and the EGFR receptor is critical for AKT phosphorylation. SMAD complex is a transcriptional activator that induces the expression of downstream target proteins. There is a variety of evidence supporting the interaction between MATN2 and EGFR to regulate BMP signalling pathways.^53^ MATN2 interaction with EGFR leads to activation of PI3K/AKT signalling and reduced synthesis of co‐binding partner SMAD4. SMAD complex acts as a transcriptional activator to induce downstream target expression and fate determination in NPCs.^38^ Therefore, exploring vascular homeostasis during embryonic brain development will help to understand the balance between neurogenesis and gliogenesis.

## AUTHOR CONTRIBUTIONS

Yuanyuan Wang conducted experiments and analysed data. Libo Su, Wenwen Wang, Jinyue Zhao, Yan Liu, Renjie Chai, Xin Li, Zhaoqian Teng, Changmei Liu and Baoyang Hu provided technical help and valuable advice. Sihan Li and Yuanyuan Wang were identified mouse genotypes. Yuanyuan Wang produced a manuscript based on comments from all the authors. Fen Ji and Jianwei Jiao overseed the project and obtained funding support.

## CONFLICT OF INTEREST STATEMENT

The authors report no biomedical financial interests or potential conflicts of interest.

## Supporting information


**FIGURE S1:** The cerebral vessels and neural progenitor cells are well‐positioned to interact in vivo and *Arid1a* is expressed in endothelial cell. (A) The blood vessels are located near astrocyte precursor cells (APCs). Immunofluorescence staining of GLAST and IB4 at E18 in the embryonic neocortex. GLAST, the astrocyte precursor cells marker; IB4, the blood vessels marker. The right shows an enlarged image of the delineated area. Scale bars, 20 μm (left), 20 μm (right). (B) The blood vessels are located near intermediate astrocytes. Immunofluorescence staining of GFAP and IB4 at P0 in the embryonic neocortex. GFAP, the astrocyte cells marker; IB4, the blood vessels marker. The right shows an enlarged image of the delineated area. Scale bars, 40 μm (left), 40 μm (right). (C) Endothelial cells were isolated from the mouse embryonic cortex. *Arid1a* was labeled with IB4 and CD31 in cultured endothelial cells and ARID1A was abundantly expressed in endothelial cells. Scale bars, 20 μm. (D) Schematic diagram of fluorescence sorting in endothelial cells. (E) Western blot analysis of ARID1A expression levels in *Arid1a* ^*fl/fl*^ and *Arid1a* ^*cKO‐Tie2*^ isolated brain endothelial cells.
**FIGURE S2.** Endothelial *Arid1a* deletion has no effect on blood‐brain barrier integrity. (A) Confocal immunofluorescence image of IB4 and platelet‐derived growth factor receptor β (PDGFRβ)show no difference in cortical coverage along the cerebral vessel (red) between *Arid1a* ^*fl/fl*^ and *Arid1a* ^*cKO‐Tie2*^. Scale bars, 20 μm. (B) Quantification of the percentage of Claudin5/IB4+ in cerebral cortex of *Arid1a* ^*cKO‐Tie2*^ with no statistical difference, *n* = 4 each group. (C) Confocal immunofluorescence images of IB4 and Claudin‐5 in *Arid1a* ^*fl/fl*^ and *Arid1a* ^*cKO‐Tie2*^ mice showed no difference in Claudin‐5, the tightly connected cerebral vessels. Scale bar, 20 μm. (D) Quantification of the percentage of Claudin5/IB4+ in cerebral cortex of *Arid1a* ^*cKO‐Tie2*^ with no statistical difference, *n* = 4 each group. (E) Confocal immunofluorescence image of IB4 and Cad‐A555 showed no cadaverine extravasation in P7 *Arid1a* ^*fl/fl*^ and *Arid1a* ^*cKO‐Tie2*^ brain cortices. Scale bars, 20 μm. Data are represented as means ± SEM. unpaired two‐tailed Student's *t*‐test; n.s., not significant.
**FIGURE S3.** Glial precursors decreased in mutant cortex. (A) Confocal immunofluorescence image of GLAST revealed a decrease in *Arid1a* ^*cKO‐Tie2*^ cortex at E18. Scale bars, 50 μm. (B) Quantification of decreased number of GLAST+ cell in *Arid1a* ^*cKO‐Tie2*^ cortex, *n* = 5 each group. (C) Confocal immunofluorescence image of GFAP revealed a decrease in *Arid1a* ^*cKO‐Tie2*^ cortex at P0. Scale bars, 20 μm. (D) Quantification of decreased number of GFAP^+^ cell in *Arid1a* ^*cKO‐Tie2*^ cortex, *n* = 5 each group. (E) Confocal immunofluorescence image of GS revealed a decrease in *Arid1a* ^*cKO‐Tie2*^ cortex at P0. Scale bars, 50 μm. (F) Quantification of decreased number of GS^+^ cell in *Arid1a* ^*cKO‐Tie2*^ cortex, *n* = 4 each group. (G) Confocal immunofluorescence image of TUNNEL revealed no difference in *Arid1a* ^*cKO‐Tie2*^ cortex at E15, Scale bars, 50 μm. (H) Quantification of no difference number of TUNEL^+^ cell in *Arid1a* ^*cKO‐Tie2*^ cortex, *n* = 3 each group. (I)Confocal immunofluorescence image of SOX2 revealed no difference in *Arid1a* ^*cKO‐Tie2*^ cortex at E13. Scale bars, 20 μm. (J) Quantification of the number of SOX2^+^ cell have no difference in *Arid1a* ^*cKO‐Tie2*^ cortex, *n* = 4 each group. Data are represented as means ± SEM. unpaired two‐tailed Student's t test, one‐way ANOVA; n.s. no significant, *p < 0.01, **p < 0.01.
**FIGURE S4.** Astrocytes are inhibited in mutant cortex. (A) Confocal immunofluorescence image of GS revealed a decrease in *Arid1a* ^*cKO‐Tie2*^ cortex at P2. Scale bars, (left) 50μm, (right) 20 μm. (B) Quantification of decreased number of GS^+^ cell in *Arid1a* ^*cKO‐Tie2*^ cortex, *n* = 4 each group. (C) Confocal immunofluorescence image of S100β revealed a decrease in *Arid1a* ^*cKO‐Tie2*^ cortex at P2. Scale bars, 50 μm. (D) Quantification of decreased number of S100β^+^ cell in *Arid1a* ^*cKO‐Tie2*^ cortex, *n* = 4 each group. (E) Confocal immunofluorescence image of NEUN revealed an increase of total neurons in *Arid1a* ^*cKO‐Tie2*^ cortex at P8. Scale bars, 50 μm. (F) Quantification of increased number of NEUN^+^ cell in *Arid1a* ^*cKO‐Tie2*^ cortex, *n* = 4 each group. Data are represented as means ± SEM. unpaired two‐tailed Student's t test, one‐way ANOVA; *p < 0.05, **p < 0.01.
**FIGURE S5.** Endothelial cells regulate the fate determination of neural progenitor cells by activating akt signaling. (A)A Kyoto Encyclopedia of Genes and Genomes (KEGG) pathway analysis was used to assess enriched pathways. The cytokine‐cytokine receptor interaction pathway was dramatically enriched (red rectangle) by RNA‐seq data analysis. (B) Immunofluorescence staining of Matn2 and CD31 in the brain cortex of *Arid1a* ^*fl/fl*^ and *Arid1a* ^*cKO‐Tie2*^ mice. Scale bars, 50 μm.(C) Knock‐down efficiency of *Shmatn2* was detected by RT‐PCR, *n* = 4 each group. (D, E) Quantification of the percent of MAP2^+^ cells and GFAP^+^ cells, *n* = 6 each group. Data are represented as means ± SEM. unpaired two‐tailed Student's *t*‐test, one‐way ANOVA; *p < 0.05, **p < 0.01, ***p < 0.0001.

## Data Availability

The RNA‐seq datasets generated and analysed during the current study have been deposited in the NCBI Sequence Read Archive (SRA). All sequencing data reported in this paper were submitted to NCBI's GEO with accession number GSE221176.
