# Supplementary material for: Endothelial Arid1a deletion disrupts the balance among angiogenesis, neurogenesis and gliogenesis in the developing brain
Source: Cell Prolif. 2023 Mar 13;56(5):e13447. doi: 10.1111/cpr.13447 (PMC10212716; doi:10.1111/cpr.13447)
Supplement: Supplementary file 1 — FIGURE S1: The cerebral vessels and neural progenitor cells are well‐positioned to interact in vivo and Arid1a is expressed in endothelial cell. (A) The blood vessels are located near astrocyte precursor cells (APCs). Immunofluorescence staining of GLAST and IB4 at E18 in the embryonic neocortex. GLAST, the astrocyte precursor cells marker; IB4, the blood vessels marker. The right shows an enlarged image of the delineated area. Scale bars, 20 μm (left), 20 μm (right). (B) The blood vessels are located near intermediate astrocytes. Immunofluorescence staining of GFAP and IB4 at P0 in the embryonic neocortex. GFAP, the astrocyte cells marker; IB4, the blood vessels marker. The right shows an enlarged image of the delineated area. Scale bars, 40 μm (left), 40 μm (right). (C) Endothelial cells were isolated from the mouse embryonic cortex. Arid1a was labeled with IB4 and CD31 in cultured endothelial cells and ARID1A was abundantly expressed in endothelial cells. Scale bars, 20 μm. (D) Schematic diagram of fluorescence sorting in endothelial cells. (E) Western blot analysis of ARID1A expression levels in Arid1a fl/fl and Arid1a cKO‐Tie2 isolated brain endothelial cells. FIGURE S2. Endothelial Arid1a deletion has no effect on blood‐brain barrier integrity. (A) Confocal immunofluorescence image of IB4 and platelet‐derived growth factor receptor β (PDGFRβ)show no difference in cortical coverage along the cerebral vessel (red) between Arid1a fl/fl and Arid1a cKO‐Tie2 . Scale bars, 20 μm. (B) Quantification of the percentage of Claudin5/IB4+ in cerebral cortex of Arid1a cKO‐Tie2 with no statistical difference, n = 4 each group. (C) Confocal immunofluorescence images of IB4 and Claudin‐5 in Arid1a fl/fl and Arid1a cKO‐Tie2 mice showed no difference in Claudin‐5, the tightly connected cerebral vessels. Scale bar, 20 μm. (D) Quantification of the percentage of Claudin5/IB4+ in cerebral cortex of Arid1a cKO‐Tie2 with no statistical difference, n = 4 each group. (E) Confocal immun [file CPR-56-e13447-s001.docx]

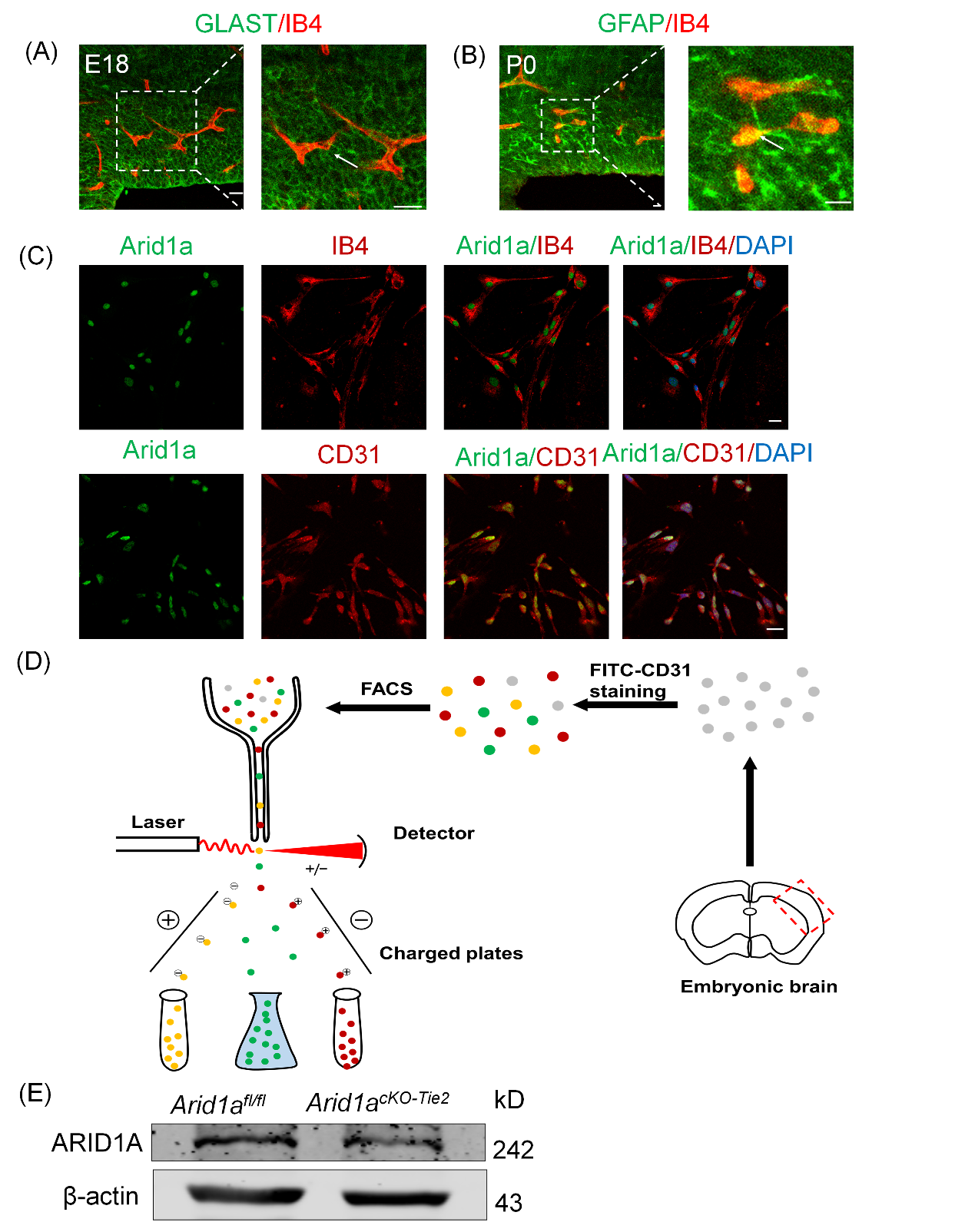


FIGURE S1 The cerebral vessels and neural progenitor cells are well-positioned to interact in vivo and *Arid1a* is expressed in endothelial cell. (A) The blood vessels are located near astrocyte precursor cells (APCs). Immunofluorescnce staining of GLAST and IB4 at E18 in the embryonic neocortex. GLAST, the astrocyte precursor cells marker; IB4, the blood vessels marker. The right shows an enlarged image of the delineated area. Scale bars, 20 μm (left), 20 μm (right). (B) The blood vessels are located near intermediate astrocytes. Immunofluorescnce staining of GFAP and IB4 at P0 in the embryonic neocortex. GFAP, the astrocyte cells marker; IB4, the blood vessels marker. The right shows an enlarged image of the delineated area. Scale bars, 40 μm (left), 40 μm (right). (C) Endothelial cells were isolated from the mouse embryonic cortex. *Arid1a* was labeled with IB4 and CD31 in cultured endothelial cells and ARID1A was abundantly expressed in endothelial cells. Scale bars, 20 μm. (D) Schematic diagram of fluorescence sorting in endothelial cells. (E) Western blot analysis of ARID1A expression levels in *Arid1a^fl/fl^* and *Arid1a^cKO-Tie2^* isolated brain endothelial cells.


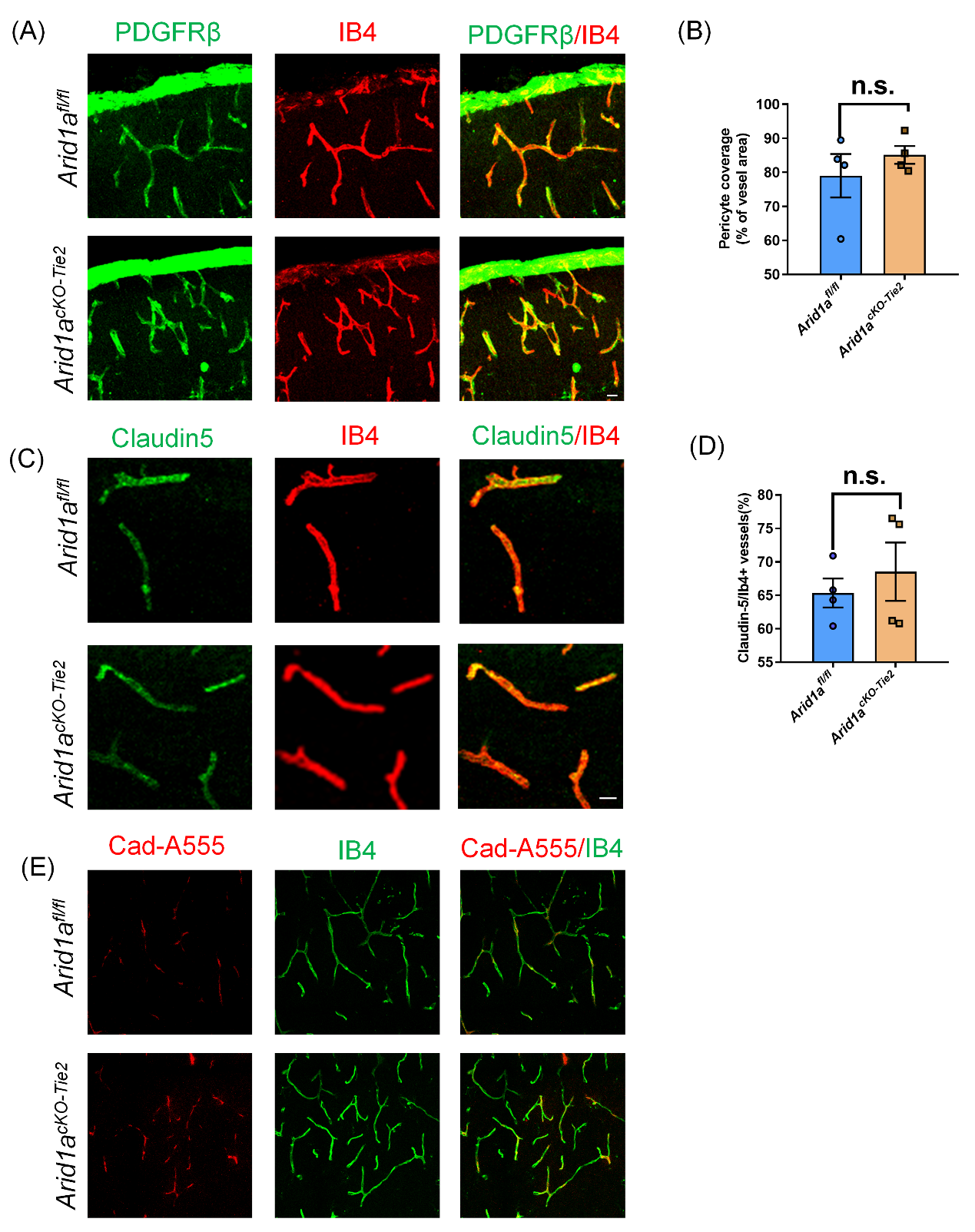


FIGURE S2 Endothelial *Arid1a* deletion has no effect on blood-brain barrier integrity. (A) Confocal immunofluorescence image of IB4 and platelet-derived growth factor receptor β （PDGFRβ）show no difference in cortical coverage along the cerebral vessel (red) between *Arid1a^fl/fl^* and *Arid1a^cKO-Tie2^*. Scale bars, 20 μm. (B) Quantification of the percentage of Claudin5/IB4+ in cerebral cortex of *Arid1a^cKO-Tie2^* with no statistical difference, n=4 each group. (C) Confocal immunofluorescence images of IB4 and Claudin-5 in *Arid1a^fl/fl^* and *Arid1a^cKO-Tie2^* mice showed no difference in Claudin-5, the tightly connected cerebral vessels. Scale bar, 20 μm. (D) Quantification of the percentage of Claudin5/IB4+ in cerebral cortex of *Arid1a^cKO-Tie2^* with no statistical difference, n=4 each group. (E) Confocal immunofluorescence image of IB4 and Cad-A555 showed no cadaverine extravasation in P7 *Arid1a^fl/fl^* and *Arid1a^cKO-Tie2^* brain cortices. Scale bars, 20 μm. Data are represented as means ± SEM. unpaired two-tailed Student’s t test; n.s., not significant.


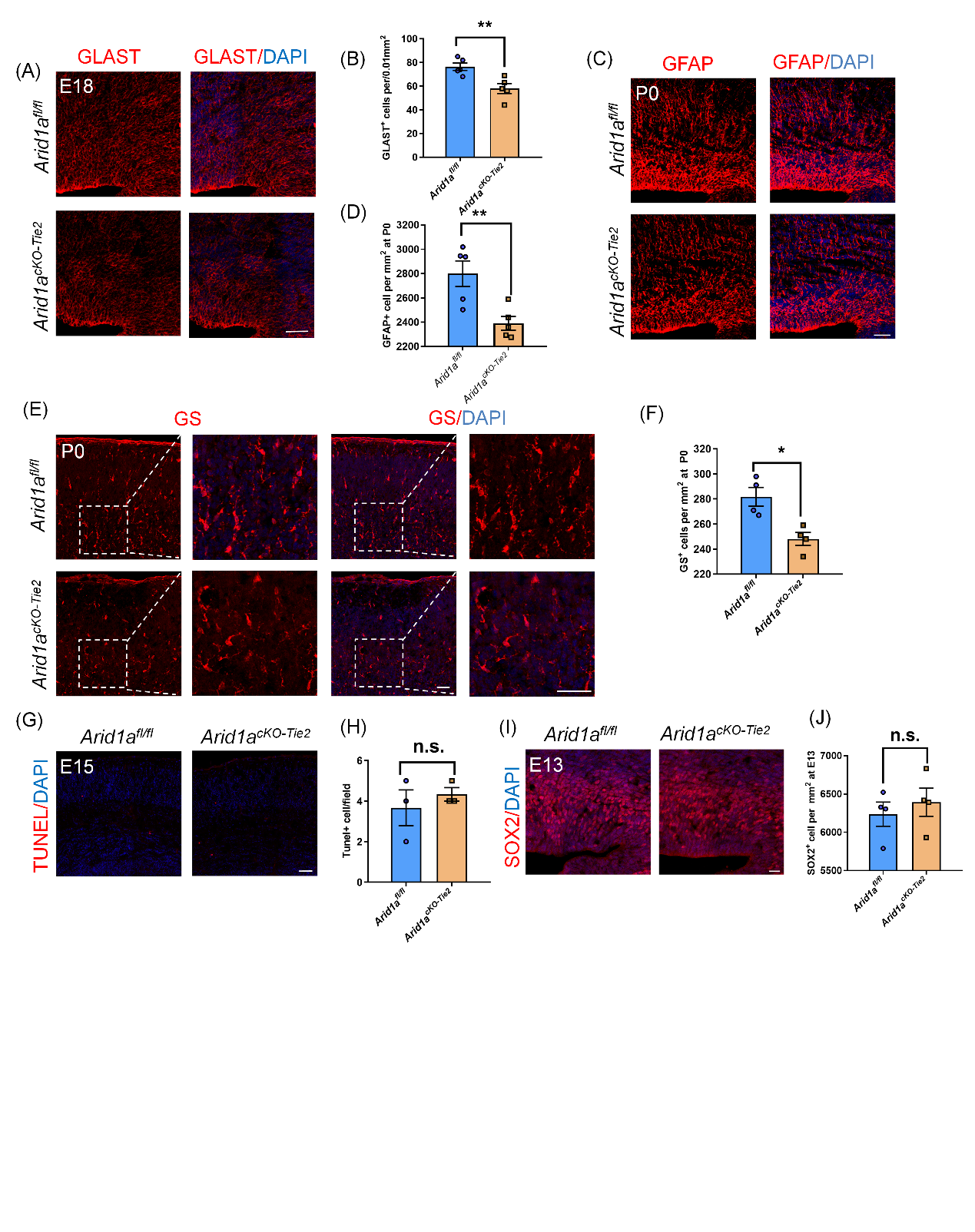


FIGURE S3 Glial precursors decreased in mutant cortex. (A) Confocal immunofluorescence image of GLAST revealed a decrease in *Arid1a^cKO-Tie2^* cortex at E18. Scale bars, 50 μm. (B) Quantification of decreased number of GLAST+ cell in *Arid1a^cKO-Tie2^* cortex, n=5 each group. (C) Confocal immunofluorescence image of GFAP revealed a decrease in *Arid1a^cKO-Tie2^* cortex at P0. Scale bars, 20 μm. (D) Quantification of decreased number of GFAP^+^ cell in *Arid1a^cKO-Tie2^* cortex, n=5 each group. (E) Confocal immunofluorescence image of GS revealed a decrease in *Arid1a^cKO-Tie2^* cortex at P0. Scale bars, 50 μm. (F) Quantification of decreased number of GS^+^ cell in *Arid1a^cKO-Tie2^* cortex, n=4 each group. (G) Confocal immunofluorescence image of TUNNEL revealed no difference in *Arid1a^cKO-Tie2^* cortex at E15, Scale bars, 50 μm. (H) Quantification of no difference number of TUNEL^+^ cell in *Arid1a^cKO-Tie2^* cortex, n=3 each group. (I)Confocal immunofluorescence image of SOX2 revealed no difference in *Arid1a^cKO-Tie2^* cortex at E13. Scale bars, 20 μm. (J) Quantification of the number of SOX2^+^ cell have no difference in *Arid1a^cKO-Tie2^* cortex, n=4 each group. Data are represented as means ± SEM. unpaired two-tailed Student’s t test, one-way ANOVA; n.s. no significant, *p < 0.01, **p < 0.01.


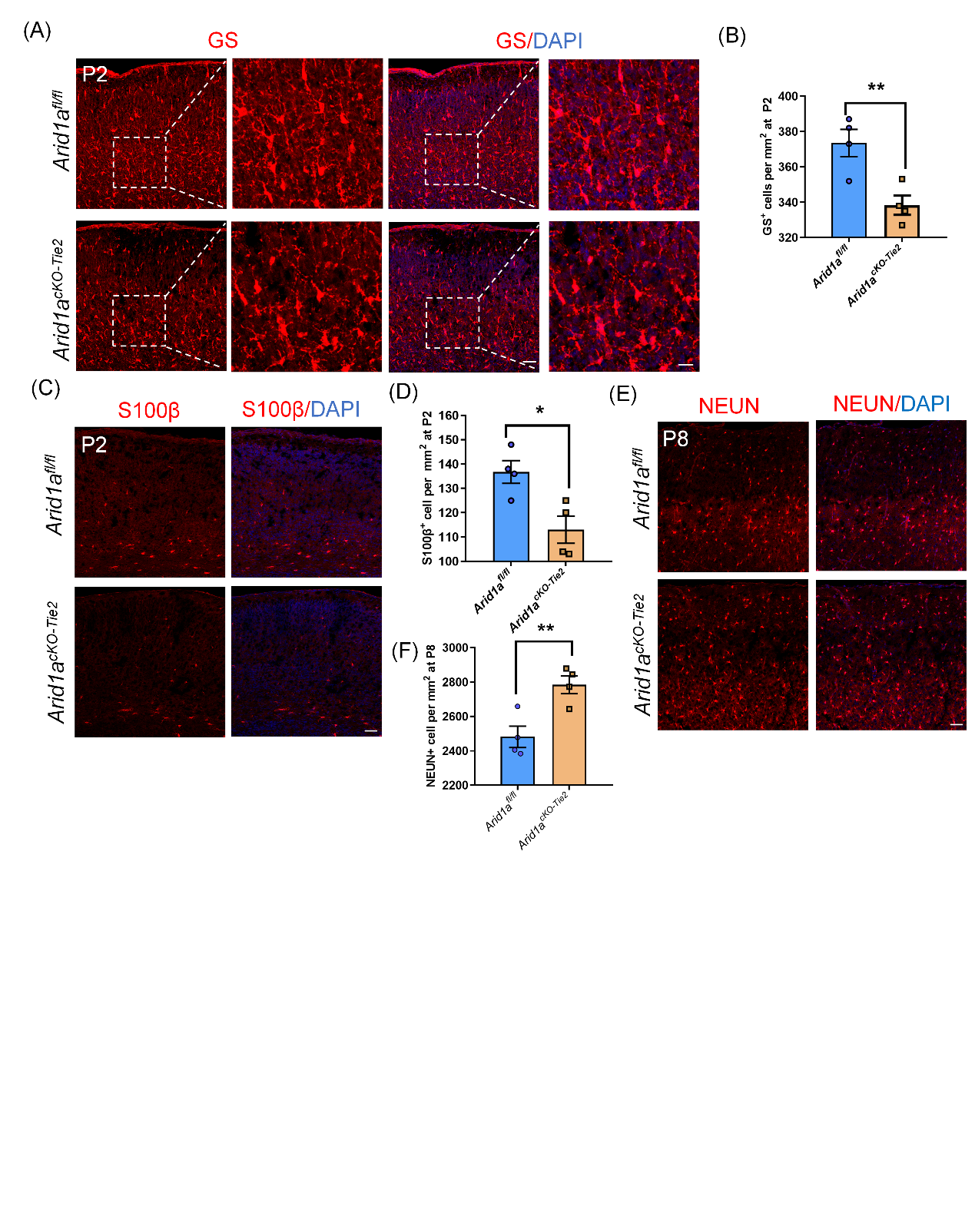


FIGURE S4 Astrocytes are inhibitied in mutant cortex. (A) Confocal immunofluorescence image of GS revealed a decrease in *Arid1a^cKO-Tie2^* cortex at P2. Scale bars, (left) 50μm, (right) 20 μm. (B) Quantification of decreased number of GS^+^ cell in *Arid1a^cKO-Tie2^* cortex, n=4 each group. (C) Confocal immunofluorescence image of S100β revealed a decrease in *Arid1a^cKO-Tie2^* cortex at P2. Scale bars, 50 μm. (D) Quantification of decreased number of S100β^+^ cell in *Arid1a^cKO-Tie2^* cortex, n=4 each group. (E) Confocal immunofluorescence image of NEUN revealed a increase of total neurons in *Arid1a^cKO-Tie2^* cortex at P8. Scale bars, 50 μm. (F) Quantification of increased number of NEUN^+^ cell in *Arid1a^cKO-Tie2^* cortex, n=4 each group. Data are represented as means ± SEM. unpaired two-tailed Student’s t test, one-way ANOVA; *p < 0.05, **p < 0.01.


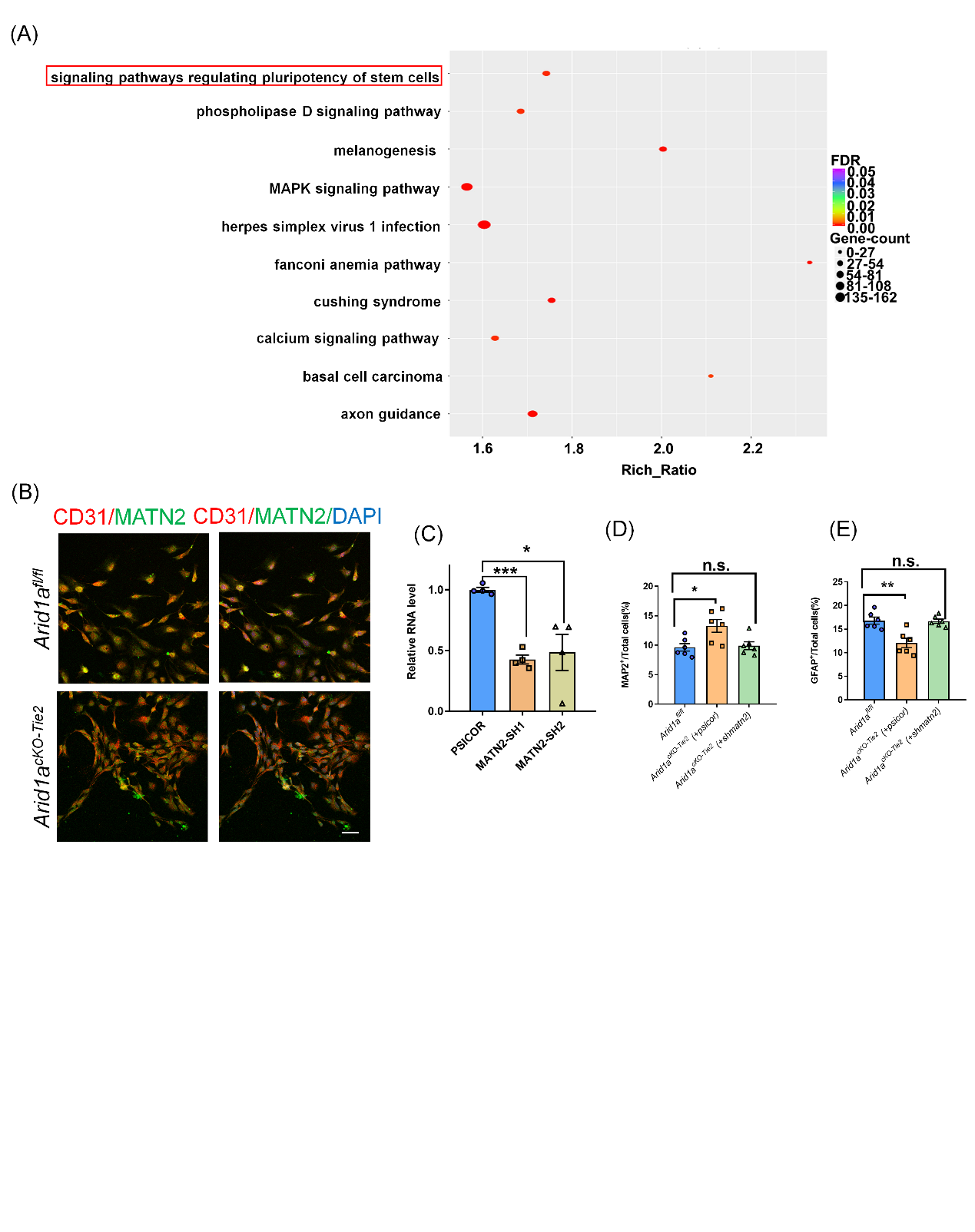


FIGURE S5 Endothelial cells regulate the fate determination of neural progenitor cells by activating akt signaling. （A）A Kyoto Encyclopedia of Genes and Genomes (KEGG) pathway analysis was used to assess enriched pathways. The cytokine-cytokine receptor interaction pathway was dramatically enriched (red rectangle) by RNA-seq data analysis. (B) Immunofluorescence staining of Matn2 and CD31 in the brain cortex of *Arid1a^fl/fl^* and *Arid1a^cKO-Tie2^* mice. Scale bars, 50 μm.(C) Knock-down efficiency of *Shmatn2* was detected by RT-PCR, n=4 each group. (D, E) Quantification of the percent of MAP2+ cells and GFAP+ cells, n=6 each group. Data are represented as means ± SEM. unpaired two-tailed Student’s t test, one-way ANOVA; *p < 0.05, **p < 0.01, ***p < 0.0001.
